# Suicide Risk in Alcohol Use Disorders: Literature Review and Study Protocol with Preliminary Data for a Study in Treatment-Seeking Inpatients

**DOI:** 10.3390/healthcare13050535

**Published:** 2025-02-28

**Authors:** Marika Krystkiewicz, Michael Soyka

**Affiliations:** 1 Fachklinik Eußerthal, Klinikstr. 1, 76857 Eußerthal, Germany; 2 Psychiatric Hospital, University of Munich, Nußbaumstr. 7, 80336 Munich, Germany

**Keywords:** suicide, suicidal behavior, alcohol, alcohol use disorder, comorbidity, meta-analyses

## Abstract

**Background/Objectives:** Individuals with substance use disorders are at risk of displaying suicidal behavior with suicide occurring more frequently compared to the general population. This article gives an overview of the existing literature on the association of substance use disorders, especially alcohol use disorder (AUD), with suicidal behavior. Studies indicate that individuals with AUD report a lifetime prevalence of 40% for at least one instance of attempted suicide. They also have a 10 to 14 times higher risk of suicidal behavior compared to the general population. **Methods:** The protocol and preliminary data from an ongoing study focused on the prevalence and clinical correlations of suicidal ideation and attempts in treatment-seeking inpatients with AUD are presented. The objective of this study is to address open questions regarding the clinical factors and psychiatric comorbidities associated with suicidality in patients with AUD. In a pilot and feasibility study in an inpatient rehabilitation facility for the treatment of substance use disorders, 150 patients with AUD were examined. For assessment, the Beck Depression Inventory (BDI-II), the Symptom Check-list-90 (SCL-90), and the Scale Suicidal Experience and Behavior (SSEV) were used as standardized questionnaires. In addition, psychosocial and sociodemographic variables were recorded. A total of 257 items were surveyed for each respondent. The statistical analysis was carried out using IBM SPSS. **Results:** The initial evaluation indicates the feasibility of the study with a high number of responders (90% response rate). Of the participants surveyed, 50.7% (N = 76) were at risk of suicide and 40.7% (N = 61) had already made at least one suicide attempt. Even with a small sample size, a significant, moderate-to-large correlation between alcohol use disorder and suicidality could be demonstrated. **Conclusions:** This confirms that suicidality is a risk factor for patients with AUD. The role of psychiatric comorbidity, clinical correlations and consequences of AUD, Gender and treatment outcome will be studied in a larger sample of 700 patients.

## 1. Introduction

### Scientific Background—State of Research

Suicide is a global challenge. In its current study of the period from 2000 to 2019, the WHO found a global decline in the suicide rate of 36%, but the global incidence was still high with a rate of 12.6 per 100,000 for men and 5.4 per 100,000 for women. Strong regional differences were found, which jeopardize the achievement of the WHO target of a further reduction in the coming years. Suicide is currently the fourth most common cause of death worldwide. One major exception in terms of reducing the suicide rate is the US. Here, the rate in 2019 was 16.1 per 100,000 inhabitants (men: 25.0; women: 7.5) compared to 12.3 per 100,000 inhabitants for both sexes, for example, in Germany (men: 18.6; women: 6.2) [1,2].

The review by Knipe et al. evaluated preventive measures for suicide and self-harm. The focus is on low- and middle-income countries, as the risk of suicide is generally higher due to special socio-economic conditions and self-harm behavior is more common, especially among women [3]. This study highlights the problem of alcohol as a contributing factor to the risk of suicide. However, it does not go into the mechanisms of action and statistical evaluations.

In their review article, Fazel et al. [4] discuss predisposing and triggering factors for suicidal behavior, among other things. Both classes of factors interact with the resilience of the individual. Predisposing and triggering factors can, in combination, lead to psychological distress that ultimately results in suicidal behavior. Drug and alcohol use disorders are considered to be strong triggers. Other triggering factors have a moderate or weak effect. Strong predisposing factors include neuropsychiatric disorder and a family history of suicidal behavior (risk factors for suicide, Table 1, page 268, [4]).

Measures that restrict access to alcohol, drugs and weapons to reduce the suicide risk also have a direct influence on the suicidal tendencies of a population [4].

Prior to the description of the ongoing clinical study, the following section gives an update on the narrative literature. We specifically address meta-analyses or cohort studies focusing on the association of suicidal behavior with substance use disorders (focus on AUD) and possible correlations and predictors of suicidality.

In recent years, a whole series of studies has provided evidence for the association between alcohol consumption and an increased risk of suicidality. The meta-analysis by Amiri and Behnezhad [5] shows that alcohol consumption increases the probability of suicidal thoughts, suicide attempts and mortality from suicide by 65%. Although a possible mutual influence of mental illnesses and their comorbidity is pointed out, it is not the direct subject of the study in this case.

A meta-analysis by Armoon B. [6] examined the relevance of variables such as mental health status, socio-demographic data and type of drug use for suicidal behavior in people with substance use disorders. Each of these variables showed significant effects on suicide risk. The results of this study also demonstrated a correlation between substance use disorders (for example, alcohol, cannabis, amphetamine, cocaine and polydrug use) and suicidal behavior. Both pure drug use disorders and combined drug and alcohol use disorders led to a significantly increased risk of suicide attempts. The odd ratios were 1.77 and 1.96, respectively. In the case of AUD alone, it was 1.58. People with SUD have a 10 to 14 times higher risk of death by suicide than the general population [6,7]. This is also consistent with the results of other studies [8]. This correlation could be explained by several mechanisms including comorbid mental illness, substance-induced depression and deterioration and disinhibition of cognitive function caused by intoxication [6].

The frequency of suicidal acts among inpatients with AUD is similar to that of people with depression, with alcohol consumption being a major risk factor for repeated suicidal acts. In psychopathological autopsy studies, it was diagnosed that 40% of those deceased from suicide suffered from a SUD. In the case of drug deaths, these figures cannot be determined precisely, as it is not always possible to distinguish between suicide and unintentional overdose. Nevertheless, the suicide rate among drug users is also high and it is assumed that about 18% of drug-related deaths are suicides. In substance users with suicidal thoughts or acts, depression is often diagnosed. In contrast to depressive disorders, suicidal acts often only occur at a late stage over the course of the addiction [9] (pp. 44–45).

To clarify the role of AUD and acute alcohol consumption in suicide attempts, Boenisch et al. [10] analyzed data on 1912 suicide attempts over a 5-year period in a major German city. The suicide attempts were categorized according to AUD diagnosis and acute alcohol consumption at the time of the attempt. In 331 suicide attempts (17%), an AUD had been diagnosed. In 622 suicide attempts (32%), acute alcohol consumption was noted. Among the individuals with AUD committing a suicide attempt, men, older individuals and individuals with a recurrent attempt were at a higher risk. This finding was independent of alcohol consumption at the time of the suicide attempt. In the case of acute alcohol consumption in suicide attempts by individuals with AUD, most often, low-risk suicide methods were chosen.

People suffering from acute alcohol intoxication also have an increased risk of death by suicide or attempting suicide [11,12,13,14]. In another meta-analysis of a total of seven studies, a possible link between alcohol consumption and suicidality was analyzed. Three case–control studies and four crossover studies were included. This study showed an overall odds ratio (OR) for suicide of 6.97 (95% CI 4.77–10.17) for any acute alcohol use (AUA) [11].

Crossin et al. [15] investigated the proportion of suicides involving acute alcohol use (AUA) among all suicides aged 15 years and older for the period from July 2007 to December 2020 (N = 1238). About a quarter of suicide deaths were related to acute alcohol intoxication. This again confirms that AUA is a significant contributor to suicidality. These New Zealand findings are consistent with international studies reporting prevalence rates for AUA of 26.5% to 44.4%.

Conner and Bagge [16] also discuss, based on meta-analyses, the role of AUD and AUA in suicidal behavior. Statistical analyses of psychological autopsies of suicide deaths provide evidence that AUD is a strong risk factor. Based on the available studies, it can be concluded that the probability of suicidal behavior is about three times higher in persons with AUD than in those without AUD. Even higher values were found based on cohort studies that looked at people with AUD undergoing treatment. This bias can be explained by the fact that this group of people exhibits more severe symptoms than the general population.

In a meta-analysis of 13 studies, Darvishi et al. [17] investigated the relationship between AUD and suicidal thoughts, suicide attempts and suicides (a study by Rossow and Amundsen was excluded in a correction). The following probabilities are reported: for suicidal thoughts OR = 1.86 (95% CI: 1.38, 2.35), for suicide attempts OR = 2.79 (95% CI: 2.16, 3.43) and for suicides OR = 2.59 (95% CI: 1.95, 3.23) RR = 1.74 (95% CI: 1.26, 2.21). The significant data used for the analyses came from cross-sectional and cohort studies.

In addition to this study, Isaacs et al. [18] conducted a meta-analysis of 33 longitudinal studies to investigate the temporal relationship or progression of AUD and completed suicides. The study confirmed that individuals with AUD have an approximately 94% increased risk of suicide. Alcohol consumption can impair emotion regulation, pain perception and social interactions, which in turn contribute to the suicide risk.

In a case–control study in two Australian states, Kõlves et al. [19] examined suicide cases with and without AUD as well as suicides and sudden deaths with AUD. The data are based on official death notifications and interviews with close relatives. It was found that people with AUD who died by suicide were significantly more likely to have other substance use disorders, a history of previous suicide attempts, partner and family problems, to have been a victim of a crime or to have a migrant background. Their average age was lower, and they had a higher potential for aggression than people who died by suicide without AUD. Individuals who died by suicide with AUD were also more likely to have mood swings, a previous suicide attempt, hopelessness, self-aggression, relationship failure, other substance use disorders or family strife than those who died suddenly with AUD. These results indicate that psychiatric comorbidity, impulsive behavior and psychosocial factors are correlated with suicidality. This confirms the need for appropriate diagnosis and risk assessment in the treatment of patients with AUD.

The main reasons for AUD being a major risk factor for suicide are its association with substance-induced depressive episodes, the disruptive impact on human relationships and recurrent intoxications with alcohol. The authors also address the gender-specific suicide risk associated with AUD. Although there is evidence that this risk has increased for women in recent years, further research on this issue is still lacking [16].

Suicide associated with alcohol use has increased in the USA from 2003 to 2018 [20,21]. It is striking that the suicide rate increased more significantly among women than among men in the same age groups. This gender-specific increase can also be seen with respect to heavy, acute alcohol consumption and alcohol use disorders. The results of the study by Lange et al. also show the interrelationship between AUD and suicidal tendencies. However, further research is needed to determine the causes of this conspicuous, gender-specific development.

The level of intoxication may be critical for suicidal behavior. In their study, Choi et al. [22] found that about one-third of all suicide deaths examined had a BAC ≥ 0.08. Bailey [23] and Ertl [24] essentially confirmed these results.

Espinet et al. [25] also pointed out in their study that it is important to examine risk and protective factors for suicidal behavior in AUD, as treatment of such patients only reduces the increased risk of suicide to a limited extent.

The probability of a first suicide attempt with a diagnosed AUD was investigated in Sweden for the birth cohorts 1950 to 1970 (N = 2,229,619) by Edwards et al. [26] in a large cohort study. The analyses were carried out on a gender-specific basis. It was found that the frequency of AUD was higher in men than in women, but the latter had a higher rate of suicide attempts. Regarding the age-dependent risk of a first suicide attempt, the following picture emerged: between the ages of 15 and 19, the risks were comparable for both genders. For women, the risk remained stable up to the age of 29 before gradually decreasing. For men, on the other hand, the risk increased up to the age of 29 and then decreased. From the age of 40, the risk remained slightly higher for both sexes (HR = 1.77 for women and HR = 1.83 for men). It can therefore be concluded that people with an early onset of AUD also have a significantly higher risk of a first suicide attempt at a young age compared to other late-onset alcohol users. Compared to the previous study by the same authors focusing on the interrelationship between AUD and completed suicide and, using the same raw data base, there were some similarities but also significant differences. The probabilities for suicide attempts were lower than for completed suicides. There were also gender-specific differences in the risk patterns. There was much less difference between the sexes in attempted suicide than in completed suicide. In the 15 to 19 age group, the risks for attempted suicide were HR = 5.5 for women and HR = 6.12 for men. However, in the same cohort, the risks for suicide were HR = 128.0 for women and HR = 28.0 for men.

In addition, Lannoy et al. [27] investigated resilience as a protective factor for suicide attempts in the presence of AUD in Sweden. For this purpose, nationwide data sets of 903,333 men born between 1960 and 1980 and 48,285 men born between 1949 and 1951 were analyzed, as resilience values were available from military records. The results showed that resilience generally provides some protection against suicide attempts. However, this effect is reduced in the presence of AUD.

Among those seeking treatment for AUD, 40% report at least one suicide attempt in their lifetime [28]. In a recent study, 4068 people with alcohol use disorder were examined, both with and without suicide attempts. In addition, genomic, clinical and neurophysiological aspects were evaluated. The results showed that people with AUD and suicidal tendencies have a higher rate of comorbid psychiatric disorders and genetically determined suicide-associated risks [28].

Høye et al. [29] investigated the mortality of inpatients and outpatients with personality disorders (N = 32,885, age 20 to 79 years, 60% women) in Norway between 2009 and 2015 based on the national patient registry regarding the interrelationship between comorbid, severe mental illness and substance use disorders. The patients were divided into the following groups:

1. Personality disorder (PD) without substance use disorder (SUD) and without severe mental illness (SMI);
2. PD with SMI without SUD;
3. PD with SUD with or without SMI.

In group 3, 55% had mixed substance use, 24% had AUD only, 12% used illegal drugs, 6% used cannabis and 4% used other unspecified/unknown drugs. A total of 890 patients died during the study period. The overall mortality of these patients was higher than that of the general population. For group 2, mortality was significantly increased compared to patient group 3. For group 3, the probability of both poisoning (standard mortality ratio 36.7) and suicide (standard mortality ratio 23.9) was very high; although, this was also the case for mortality from natural causes for group 3. The results show that SUD and comorbidity have an impact on the mortality rate [29].

A similar study [30] investigated the relationship between suicide attempts and remitted substance use disorder, evaluating comorbidity and psychosocial factors in 354 patients with SUD in remission who had made at least one suicide attempt. Next, 135 individuals with a lifetime DSM diagnosis of SUD were compared with 219 patients without a lifetime diagnosis of SUD. The group with lifetime SUD was more likely to be male and less educated. However, the two groups did not otherwise differ in terms of socioeconomic factors, comorbid anxiety disorders or eating disorders. The group with lifetime SUD was more likely to be in residential treatment and to meet the criteria of a cluster B personality disorder at the time of the study. The two groups did not differ regarding other personality disorders. In total, 80% of the lifetime SUD group had a diagnosis of AUD, 11.1% were benzodiazepine users, 27.8% were cannabis users, 34.1% were stimulant users (including cocaine) and 3.7% were hallucinogen users. Also, 48.1% had a lifelong dependence on at least two substances.

AUD is generally associated with a wide range of additional disorders such as increased aggression, violence, accidents and suicidal tendencies. However, neuropsychiatric and physical illnesses can also be associated with it [31,32]. Cognitive impairment resulting from chronic alcohol consumption can reduce the ability to make adequate decisions, regulate emotions and control one’s own behavior and consequently results in an increased risk of suicidal behavior [33].

Buri et al. [34], in the German-speaking part of Switzerland, examined predictive factors for suicide attempts in alcohol-dependent patients. They propose a decision tree that can be used to facilitate the identification of AUD patients with high suicide risk. A sample of 700 patients (34.7% women) was obtained from a total of 12 treatment programs for patients with AUD in a retrospective, multi-center study. A total of 69 (9.9%) of these patients reported a suicide attempt in the last 3 months before starting treatment. Receiver operating characteristic (ROC) analyses were used to identify patients who were at risk of having attempted suicide in the last 3 months.

Regarding the influence of AUA on suicidal behavior, Conner et al. [16] cite the following mechanisms that can increase the risk of suicide: psychological stress, depressive mood, aggression, impulsivity and the impairment of cognitive function under the influence of alcohol. In addition, studies show an increase in suicidal thoughts during alcohol intoxication, which in turn can lead to an impulsive suicidal act. Between a quarter and a third of the people surveyed stated that the motives for their acute alcohol consumption before the suicide attempt were to gain courage, suppress fears and numb fears or anesthetize the pain of dying. The simultaneous consumption of other substances that affect the central nervous system can significantly increase the likelihood of suicide attempts.

A previous study [35] of 182 alcohol-dependent, detoxified patients with a history of at least one suicide attempt showed that they were more likely to exhibit aggressive and impulsive behavior. The rate of depressive disorders was also high in these patients.

In a study of 304 alcohol-dependent patients, Jakubczyk et al. [36] were able to find statistical evidence of a relation between the level of cognitive impulsivity and the severity of depression. Other authors [30,37,38] also point to the link between alcohol consumption and an increase in impulsive behavior.

Pichler and Walter [39] suggest that correlating factors in alcohol use disorder—such as impulsivity or genetic predisposition—may be the cause of the link between suicide and comorbidity.

In experimental settings under laboratory conditions in people with mood disorders, the release of cortisol under the influence of stress was investigated by Stanley et al. [40]. The patients were divided into two groups: people with (N = 35) and without (N = 37) suicide attempts. The group with suicide attempts was further clustered according to their level of aggression and impulsiveness. It was found that under the influence of stress, the release of cortisol was higher in people with suicide attempts than in the control group, particularly in people with high levels of impulsivity and aggression.

Other studies have shown clear correlations between chronic and acute alcohol consumption and reduced sensitivity to pain. However, further studies are needed to determine the interrelationship between pain, alcohol’s analgesic effect and the risk of self-harm/suicide [41,42]. Relapses to alcohol consumption in abstainers with AUD often cause feelings of helplessness and a decline in self-efficacy and control. Consequently, suicidal ideations may emerge [43] (p. 94).

In a German cohort study [44] from three independent population-based cross-sectional surveys conducted in 1984/85, 1989/90 and 1994/95 with a total of 12,888 subjects aged 25–74 years, standardized mortality ratios (SMRs) were calculated for deaths by suicide in connection with smoking and high alcohol consumption. Compared to the general population, suicide mortality was higher for risky alcohol consumption (SMR = 2.37; 95% CI 1.14–4.37) and smoking (SMR = 2.30; 95% CI 1.36–3.63). A significant increase in suicide mortality (SMR = 4.80; 95% CI 2.07–9.46) was observed in smokers with risky alcohol consumption. In these individuals, a fourfold increased risk of suicide was found compared to the general population.

In a very recent study in 2024 by Grote et al. [45], women with existing SUD and suicidal thoughts responded significantly better than men to treatment interventions to reduce suicidality. For them, the frequency of suicidal thoughts was reduced from 21.7% before treatment to 7.7% after treatment. For men, this ratio is 16.7% to 11.6%.

In the study by Pavarin et al. [46], statistically significant evidence was found that patients with substance use disorders who required acute clinical help between 2009 and 2019 had a reduced risk of suicidal behavior in the first year of treatment. The patient cohort consisted of 2874 people and comprised 53% cocaine-, 46% opiate- and 35% cannabis-dependent patients. In total, 31% had concomitant AUD and 30% had a comorbid psychiatric disorder. Since the statistical analyses were partly covering the COVID-19 pandemic during the lock-down phase, too few data points were available from this period to evaluate the influence of the pandemic on suicidal behavior [46].

The dampening effect of sobering up on acute suicidal behavior also plays a role in suicide prevention [47].

In conclusion, targeted therapeutic interventions to reduce alcohol consumption could help to curb suicidal behavior. However, sufficient systematic investigations and meta-analyses are still lacking to assess the effect of alcohol-specific interventions on the suicidal behavior of individuals. A meta-analysis of 11 studies, 9 of which were statistically significant, showed the positive effect (albeit non-significant) of psychotherapeutic interventions on suicidal ideation and self-harm. Due to insufficient data, no definite conclusions could be made about the effect on suicides [48].

Suicide rates clearly vary between countries. In a study [49,50], the German Institute for Economic Research (DIW) showed a decrease in deaths from suicide related to alcohol and drug use in Germany, while they have increased in the USA over the same period. For the development in the USA, they referred to two studies by Case and Deaton [51,52], which are based on data from the cause-of-death statistics and population updates. They show that mortality rates among non-Hispanic whites aged 50 to 54 increased in the USA since the end of the 1990s. This was attributed to an increasing number of suicides and deaths from drug overdose or alcohol-related liver disease, particularly among people with low levels of education. According to Case and Deaton, this may be explained by a change in living conditions, for example, due to fewer chances in the labor market or increasingly unstable family situations (deaths of despair). This contrasts with the trend in Germany for the population aged between 50 and 54. Here, mortality from suicides and deaths related to alcohol and drug use fell between 1990 and 2015. This applies both in terms of gender and place of residence in western or eastern Germany. However, a difference in nationality and marital status also shows a decline in rates. The results for eastern Germany are particularly noteworthy because the economic and social living conditions of people there changed dramatically during the period under review following the reunification of Germany in 1990. The authors discuss possible reasons for the differences found between the USA and Germany with respect to different welfare systems.

In 2014, Lithuania was the country with the third highest level of alcohol consumption in the world. Per capita consumption of alcohol was more than 15 liters in persons aged 15 and above [53]. Due to strict regulations this amount fell to 11 liters in 2023 [54]. So, alcohol consumption decreased by 26.7% between 2014 and 2023.

Also, in 2015, Lithuania still was the country in Europe with the highest rate of suicides and the country with the fifth highest rate worldwide (28.2/100,000 inhabitants). The age-adjusted suicide rates for male and female subjects were 51.0 and 8.4 per 100,000 inhabitants [53]. By the end of 2022 these figures had declined to a total rate of 18.6/100,000, 32.2/100,000 for males and 6.7 for females [55]. Thus, the suicide rate had dropped by 34% between 2015 and 2022.

Between June 2015 and July 2016, Dambrauskiene et al. [53] conducted a study in Kaunas, which included a total of 561 suicide attempters being hospitalized after their attempt. The objective of the study was to analyze the association between a suicide attempt and drinking patterns. It also evaluated risk factors for suicide attempts among problem drinkers versus non-problem drinkers. The statistical evaluation showed a significant association between alcohol consumption and suicide risk. In total, 70.9% of the male and 43.2% of the female suicide attempters were problem drinkers. The suicide risk was 3.2 times higher for males, 1.08 times for younger people, 1.58 times for people married or in a partnership, 2.04 times for people with less than 12 years of formal education and 8.15 times for people with acute alcohol intoxication.

**Table 1:** Key findings on suicidality.

Findings	Source
General suicide risk: 16.1/100,000 suicides (USA); 12.3/100,000 suicides (Germany).	[2]
On AUD 65% increase in suicidality risk.	[5]
Significant increased risk of suicide attempts. Odds ratio 1.58 on AUD. Odds ratio 1.77 on SUD. Odds ratio 1.96 on AUD + SUD.	[6]
On AUD + SUD, 10 to 14 times increased risk of death by suicide.	[6,7]
Suicides of people with personality disorders (PD). With AUD only ~24% of sample. AUD + SUD ~55% of sample. PD+SUD with/without severe mental illness SMI: significant increase in risk. PD+SMI, without SUD: increased risk.	[29]
AUD + SUD increases suicide risk by 40%.	[9]
Suicide attempts with AUD diagnosis: frequency of 17%. Suicide attempts with acute alcohol consumption: frequency of 32%.	[10]
Odds ratio for suicide of 6.97 for any acute alcohol use.	[11]
Circa 25% of suicide deaths are related to acute alcohol intoxication. International prevalence rates for AUA of 26.5% to 44.4%.	[15]
Relationship between AUD and suicidality. Odds ratio 1.86 for suicidal thoughts. For suicide attempts: 2.79. For suicides: 2.59.	[17]
Approx. 94% increase in completed suicides of people with AUD.	[18]
About 1/3 of suicide deaths with blood alcohol content (BA)C ≥ 0.08.	[22,23,24]
Probability of a first suicide attempt with diagnosed AUD. Frequency of AUD higher in men than in women. Higher rate of suicide attempts in women. Early onset of AUD correlates with significantly higher risk of first suicide attempt at young age.	[26]
People seeking treatment for AUD report frequency of 40% for suicide attempt.	[28]

Based on the above-mentioned studies, it is evident that several data sets from international publications are available regarding the interrelationship between alcohol use disorder and an increased risk of suicidal behavior. There definitely are cultural and demographic differences for the regions covered by the above studies. Therefore, the results given may not be generalized. Nevertheless, international studies show that people with AUD represent a high-risk group for suicidal behavior. Unfortunately, there are only a limited number of quantitative studies on SUD, suicidality, co-morbidity, and psycho-social factors available especially in German-speaking countries.

In our ongoing clinical study, we are investigating the prevalence of suicidal tendencies and their clinical correlations in a large group of treatment-seeking individuals with AUD. This study has several objectives:

1. To show the feasibility and practicability of the research protocol.
2. To assess and quantify the prevalence of suicide attempts in treatment-seeking patients with AUD.
3. To assess clinical correlations and predictors of suicidality in patients with AUD.
4. To investigate the association of a history of suicide attempts with treatment outcomes in a standardized treatment program.
5. To evaluate possible gender differences concerning a risk of suicide.
6. To address the relevance of suicide attempts for treatment outcomes in this setting.

## 2. Materials and Methods

### 2.1. Research Design and Methodology

As part of a single-center (monocentric), quantitative, and anonymous cross-sectional study of patients with AUD in a publicly funded inpatient rehabilitation clinic, questionnaires have been used from March 2023 to investigate the correlation between AUD, comorbidity and suicidality. The duration of treatment in the rehabilitation facility is usually 13 weeks. For the study, the largest possible study population will be recruited from the clinic’s patient pool to obtain statistically relevant results. It is planned to survey a total of around 700 patients (N = 700). Before the start of the study, the minimum sample size was estimated. The following assumptions were made: The possible total patient pool comprises 720 people during the survey phase (total capacity of clinic). A confidence level of 95%, a Z-value of 1.96 and a margin of error of 5% were assumed. This results in a minimum sample size of 251 patients for the full-scale study.

To determine the minimum required sample size for correlations (bivariate normal model), the following assumptions were made during an A priori analysis. A correlation coefficient of 0.3 (moderate correlation), a significance level of 0.05 and a statistical power of 0.95 were assumed. This resulted in a minimum sample size of 138 [56,57]. A sensitivity analysis was carried out for the full-size study with an expected sample of N = 700—with otherwise unchanged parameters. According to this, the correlation coefficient may only be 0.136 (weak correlation) for the correlation to be proven as significant [56,57].

The study was granted ethical and legal approval by the Ethics Committee of the Ludwig Maximilian University Munich on 9 January 2023.

### 2.2. Recruitment

Study participants are typically recruited between the 1st and 4th week of inpatient treatment. Each patient is invited to an informative screening assessment. During this interview, the patient is invited to participate in the study. Information is provided about the purpose and content of the survey, followed by a declaration of consent and participant information. Patients can address any unanswered questions regarding participation in the study. After completion of the recruitment interview, the survey is conducted using the standardized instruments described below plus a questionnaire specially developed for the study. After the data collection, a debriefing is conducted with the participants to identify any signs of an impending or acute crisis and to be able to intervene.

### 2.3. Study Population (Inclusion/Exclusion Criteria, Recruitment, Data Collection)

The following inclusion and exclusion criteria are used to recruit patients for the study.

Inclusion criteria

- Age 18 years or above at the start of treatment;
- Patients diagnosed with substance use disorder as first diagnosis according to ICD-10 criteria [58];
- Completed detoxification, no withdrawal symptoms.

Exclusion criteria

- Patients with no capacity for rehabilitation (acute suicidal tendencies requiring emergency treatment, intoxication, acute mental and physical disorders);
- Lack of informed consent;
- Cognitive impairment;
- Insufficient language skills (writing and verbal).

### 2.4. Instruments

The following instruments were used to collect the data. As standardized questionnaires, the Beck Depression Inventory (BDI-II) [59,60], the Symptom Checklist-90 (SCL-90) [61] and the Scale Suicidal Experience and Behavior (SSEV) [62], as well as a self-developed questionnaire. A total of 257 items were given for each respondent. Likert scales as well as binary options (yes/no) were used. The items comprise grouped questions on the subject’s areas of personal data, specific questions on suicidal tendencies, the family, professional and health situation and the respondent’s risk factors. The individual’s history of substance use, suicidality and psychiatric comorbidity was recorded.

### 2.5. Statistical Methods for Future Data Analysis

Regression analyses were carried out to evaluate the data collected after the patient surveys were completed. This phase is essentially divided into the following steps: A plausibility check and a selection of valid questionnaires is carried out and the handling of missing data is clarified. The subsequent data collection and, if necessary, evaluation is carried out with a statistical software package (IBM SPSS). The minimum statistical power to be achieved for the regression tests will be calculated. In the validation phase of the statistical model, it will be ensured that the model reflects the underlying relationship and was not created by random effects. The final interpretation of the results will then be used to prepare and present statistically proven or disproven correlative relationships.

## 3. Results

### Preliminary Results

An initial sample of 150 participants (N = 150) was examined and descriptive statistical evaluations were carried out. In this first phase of the survey, the response rate was about 90%. The proportion of male patients was 74% (N = 111), 25.3% (N = 38) female and 0.7% diverse (N = 1). The male study participants had an average of 44 years, while the female participants were 49 years old. Regarding the duration of dependence, the following picture emerged: about 25% had been dependent for 5 or fewer years, 16% for 6 to 9 years and around 59% for 10 or more years.

With a frequency of 50.7% (N = 76) the participants surveyed were at risk of suicide and 40.7% (N = 61) had made at least one suicide attempt (see Table 2).

Possible correlations were also looked at. First, the self-assessment regarding the statement “Due to my addiction, I am more at risk of suicide than people without addiction” was checked against suicide risk as determined by standardized instruments. For this purpose, a binary suicidal tendency value was determined from several indicator values of the standardized instruments and then used to examine the correlation. The results showed a significant positive correlation of 0.308 (see Table 3).

The same question on self-assessed suicide risk was also tested against the self-assessment “I fear that I will attempt suicide in the future”. This resulted in a somewhat stronger Pearson correlation of 0.33 (see Table 4).

## 4. Discussion

Data from the narrative review clearly indicate a significant association of suicide and suicidal behavior and AUD. There are still open questions, especially on clinical correlations and predictors of suicidal behavior in AUD, its relation to treatment outcome and the efficacy of targeted interventions. In this large cross-sectional study, the prevalence of suicide attempts in treatment-seeking patients with AUD is studied. To date, a total of 150 subjects took part in this study. The participation rate of about 90% is high and demonstrates the feasibility of the study. The refusal rate prior to the start of the survey is about 10%. Reasons given by patients for not participating in the study included, for example, poor German language skills or illiteracy, lack of concentration or interest or fear of recalling past suicidal behavior. A detailed analysis of the reasons, their frequency, and distribution is not possible due to the data protection rules applicable in Germany. Since there were no dropouts during the actual survey, the practical feasibility of the questionnaires (both the standardized and the self-developed ones) can be stated. For this study, a set of standard instruments is used together with a questionnaire focusing on psychosocial parameters. For daily use in clinical practice, the large number of items surveyed (N = 257) in the current study may not be applicable, although the patients in this study only needed between 25 and 45 min to answer all the questions. For clinical use, however, a more compact instrument could be developed focusing on key items.

The preliminary results show that both suicidal tendencies and comorbidity are very frequent in people with AUD. A substantial number of patients reported suicide attempts (40%) and ideas in the past. The results obtained from the initial 150 participants surveyed correlate with figures cited by Bronisch [9] and Barr [28]. Psychosocial factors and correlations of all relevant variables will be evaluated after the completion of data acquisition. For this, indicator values of the survey sections on suicidality, social and professional environment, self-image, problem-solving skills, financial situation and social support will be combined into suitable variables and used for correlation. Possible predictors of suicidal ideas will be assessed after the completion of the survey. In addition, the relationship between a history of suicidal behavior and attempts and treatment outcome (retention rate, relapse during treatment) will be evaluated. Once the study has been completed, it may be possible to answer some open questions regarding the prevalence of suicidal thoughts and behavior in German-speaking patients with AUD and to develop more focused clinical instruments for early detection and intervention in people with AUD and suicidal ideations.

## 5. Limitations

This is a non-interventional pilot study. The current findings are preliminary but indicate the feasibility of the study. The significance of this study is limited by the fact that it is restricted to just one rehabilitation clinic with inpatients. A generalized causal relationship between AUD and the risk of suicide cannot be derived from these data. This would require a longitudinal study. Furthermore, the data and clinical history are self-reported, as in most other studies on this issue. The strength of the study includes the large target number of participants (N~700) and the high number of items assessed.

To reduce the probability of type 1 errors, the full-sized study will rely on a sufficiently large sample size in combination with an alpha level of 0.05 or less. To address the risks of such errors in multiple comparisons, Bonferroni corrections will be applied.

Due to the cross-sectional study design and the lack of a second survey of the same participants or a long-term follow-up, no data on changes in suicidal ideation in and beyond the initial treatment can be derived. Any follow-up study should address this issue.

The ethnic and linguistic heritage of the participants is not recorded in this study. A follow-up study in a broader clinical setting should also record and investigate the cultural background.

## 6. Conclusions

Multiple lines of evidence show a significant association between alcohol use disorders and the risk of suicide. The goal of this ongoing non-interventional cross-sectional clinical study is to further investigate the interrelationship between alcohol use disorders and suicidality and the identification of risk factors, with a focus on comorbid psychiatric disorders. In addition, the role of psychosocially relevant factors is assessed. To date, even with a small sample size, a moderate-to-high correlation between AUD and suicidality could be demonstrated.

Further research in this area can contribute to the development of reliable instruments for risk assessment and treatment approaches for substance users with suicidal tendencies. The results of the current study may contribute to improving early detection of individuals at risk. In clinical practice, suicidality should be given greater attention in patients with SUD. This requires adequate therapeutic and preventive measures as well as an awareness of the social environment to reduce the potential suicide risk.

## Figures and Tables

**Table 2:** Frequency of suicide attempts among the respondents.

In My Lifetime I Tried to Kill Myself (and I Really Wanted to Die)					
		Frequency	Percentage	Valid Percentage	Cumulated Percentage
Valid	No	89	59.3	59.3	59.3
	Yes	61	40.7	40.7	100
	Total	150	100.0	100.0	

**Table 3:** Correlations between dependence, self-assessment of suicidal risk and statistically determined suicidal risk.

Correlations			
		Due to my Use Disorder, My Suicide Risk Is Higher Than That of People Without a Use Disorder	Suicide Risk
Due to my use disorder, my suicide risk is higher than that of people without a use disorder	Pearson correlation	1	0.308 **
	Sig. (2-sided)		<0.001
	N	144	138
Suicide risk	Pearson correlation	0.308 **	1
	Sig. (2-sided)	<0.001	
	N	138	140

** The correlation is significant (2-sided) on the level of 0.01.

**Table 4:** Correlations between dependence, self-assessment of suicide risk and self-assessed probability of a future suicide attempt.

Correlations			
		Due to My Use Disorder, My Suicide Risk Is Higher Than That of People Without a Use Disorder	I Am Afraid that I will Attempt Suicide in the Future
Due to my use disorder, my suicide risk is higher than that of people without a use disorder	Pearson correlation	1	0.330 **
	Sig. (2-sided)		<0.001
	N	144	144
I am afraid that I will attempt suicide in the future	Pearson correlation	0.330 **	1
	Sig. (2-sided)	<0.001	
	N	144	146

** The correlation is significant (2-sided) on the level of 0.01.

## Data Availability

Data is contained within the article.
